# Inhalation injury in severely burned children does not augment the systemic inflammatory response

**DOI:** 10.1186/cc5698

**Published:** 2007-02-16

**Authors:** Celeste C Finnerty, David N Herndon, Marc G Jeschke

**Affiliations:** 1 Shriners Hospitals for Children and Department of Surgery, University of Texas Medical Branch, 815 Market Street, Galveston, TX, USA

## Abstract

**Introduction:**

Inhalation injury in combination with a severe thermal injury increases mortality. Alterations in inflammatory mediators, such as cytokines, contribute to the incidence of multi-organ failure and mortality. The aim of the present study was to determine the effect of inhalation injury on cytokine expression in severely burned children.

**Methods:**

Thirty severely burned pediatric patients with inhalation injury and 42 severely burned children without inhalation injury were enrolled in the study. Inhalation injury was diagnosed by bronchoscopy during the first operation. Blood was collected within 24 hours of admission and again at five to seven days following admission. Cytokine expression was profiled using multi-plex antibody-coated beads. Significance was accepted at a *p *value of less than 0.05.

**Results:**

The mean percentages of total body surface area burned were 67% ± 4% (56% ± 6%, third-degree burns) in the inhalation injury group and 60% ± 3% (45% ± 3%, third-degree burns) in the non-inhalation injury group (*p *value not significant [NS]). Mean age was 9 ± 1 years in the inhalation injury group and 8 ± 1 years in the non-inhalation injury group (*p *value NS). Time from burn to admission in the inhalation injury group was 2 ± 1 days compared to 3 ± 1 days in the non-inhalation injury group (*p *value NS). Mortalities were 40% in the inhalation injury group and 12% in the non-inhalation injury group (*p *< 0.05). At the time of admission, serum interleukin (IL)-7 was significantly increased in the non-inhalation injury group, whereas IL-12p70 was significantly increased in the inhalation injury group compared to the non-inhalation injury group (*p *< 0.05). There were no other significant differences between groups. Five to seven days following admission, all cytokines decreased with no differences between the inhalation injury and non-inhalation injury cohorts.

**Conclusion:**

In the present study, we show that an inhalation injury causes alterations in IL-7 and IL-12p70. There were no increased levels of pro-inflammatory cytokines, indicating that an inhalation injury in addition to a burn injury does not augment the systemic inflammatory response early after burn.

## Introduction

During the past 20 years, mortality from major burns has decreased due to improved intensive care unit care, improvements in wound management, better control of sepsis, and control of hemodynamic disorders [1,2]. Of the injuries now associated with burns, the single most important contributor to mortality is inhalation injury. Twenty to thirty percent of all major burns are associated with a concomitant inhalation injury and a mortality of 25% to 50% when patients required ventilator support for more than one week following injury [2].

Lung injury from smoke inhalation is associated with tracheobronchial hyperemic sloughing of ciliated epithelium, formation of copious tracheal exudates, and pulmonary capillary permeability changes that result in a pulmonary edema [3]. Further studies show a progressive increase in lung permeability soon after thermal injury [4]. The inhalation of toxic smoke causes the release of thromboxane and other mediators, which increases pulmonary artery pressure and causes secondary damage to the respiratory epithelium and release of chemotactic factors [3]. Neutrophils subsequently undergo diapedeses from the pulmonary microvasculature and release enzymes such as elastase and free oxygen radicals, disrupting endothelial junctions and the epithelial integrity, thus permitting an exudate of protein-rich plasma to enter the lung [3]. A concomitant reduction in the pulmonary immune function may lead to bacteria growth and pneumonia [5].

The pathophysiology of smoke inhalation injury has been well studied; however, the molecular and cellular mechanisms are still not entirely known. We hypothesized that the systemic inflammatory response plays an important role in the clinical aftermath of an inhalation injury. The systemic inflammatory response to burn encompasses the release of large quantities of cytokines such as interleukin (IL)-1β, IL-6, IL-8, or tumor necrosis factor (TNF) [6-10]. Anti-inflammatory cytokines such as IL-2, IL-4, or IL-10 are released concurrently in an attempt to counter-regulate the effects of pro-inflammatory cytokines [10]. Elevation of pro- and anti-inflammatory cytokines alters immune function and protein metabolism, and these alterations can lead to compromise of the structure and function of multiple organ systems [6,11-14]. Hypermetabolism also leads to futile protein use, resulting in induction of a dynamic hypercatabolic state [15-18]. These findings delineate the importance of cytokines as pro-inflammatory mediators. The aim of the present study was to determine whether an inhalation injury further augments the inflammatory response after a severe burn injury, contributing to increased mortality via the altered inflammatory response.

## Materials and methods

### Patients

**Figure 1:**
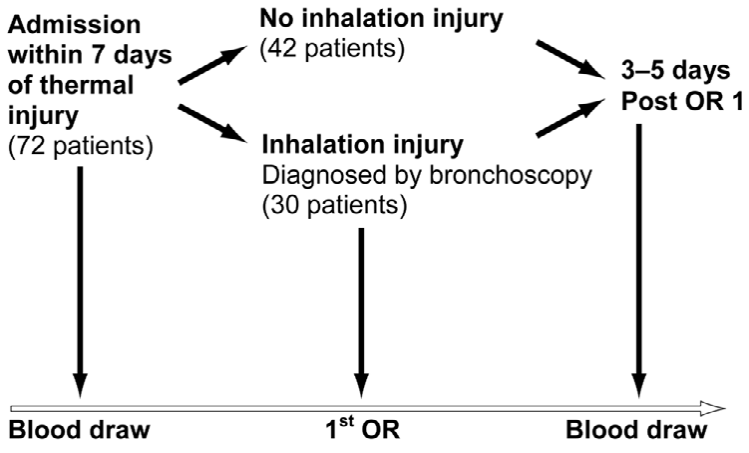
An outline of the study. To participate in the study, the patient had to be admitted to our institute within seven days after the burn injury. Patients were usually taken to the operating room (OR) within 48 hours after admission. Following evaluation of clinical signs and bronchoscopy findings, patients were then divided into control patients and those with inhalation injury. Blood was drawn at admission and three to five days after the first surgery.

Thirty severely burned children suffering from inhalation injury and 42 severely burned children without inhalation injury were enrolled in this prospective study (Figure 1). Inclusion criteria were age of 16 years or younger, admission within seven days after injury to the Shriners Hospitals for Children-Galveston (Galveston, TX, USA), and burns covering more than 40% of total body surface area (TBSA) with a third-degree component of more than 34% requiring at least one surgical intervention for escharotomy and skin grafting. Patients were excluded if there was any sign of infection or sepsis or concomitant major injuries or complications at admission.

After admission, patients were treated according to the standard of burn care at our institute, including early excision and grafting of the burn wound and fluid and caloric resuscitation according to the Galveston formulas [11]. Patients were fed enterally due to our findings that total parenteral nutrition is associated with higher mortality [19].

Diagnosis of inhalation injury was made by bronchoscopy during the first operation, which usually occurs within 24 hours after admission. An experienced anesthesiologist and/or respiratory therapist performed the bronchoscopy and made the diagnosis of inhalation injury based on the following: (a) Clinical criteria were history of exposure to smoke in a closed space (for example, patients who were stuporous or unconscious) and presence of facial burns, singed nasal vibrissae, bronchorrhea, sooty sputum, or auscultatory findings such as wheezing or rales. (b) Laboratory criteria were hypoxemia and/or elevated levels of carbon monoxide. (c) Bronchoscopy criteria were airway edema, inflammation, mucosal necrosis, presence of soot and charring in the airway, tissue sloughing, or carbonaceous material in the airway.

**Figure 2:**
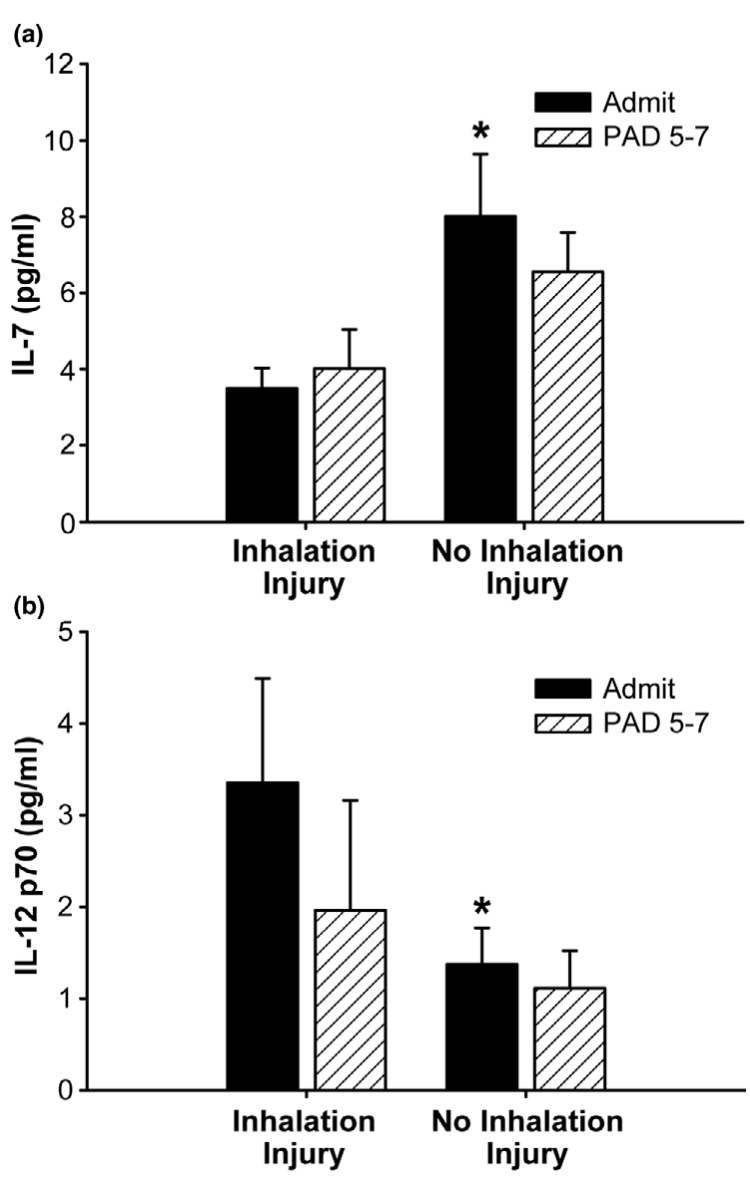
Two cytokines were significantly different between patients with no inhalation injury and those with an inhalation injury, namely interleukin (IL)-7 and IL-12p70. **(a) **IL-7 was significantly increased in the group with no inhalation injury compared to the inhalation injury group. Normal IL-7: 3.8 ± 0.63 pg/ml. **(b) **IL-12p70 was significantly decreased in the group with no inhalation injury compared to the inhalation injury group. Normal IL-12 p70: 0 ± 0 pg/ml. *Significant difference between inhalation injury group and group with no inhalation injury (*p *< 0.05). Data are presented as mean ± standard error of the mean. PAD, post-admission day.

**Figure 3:**
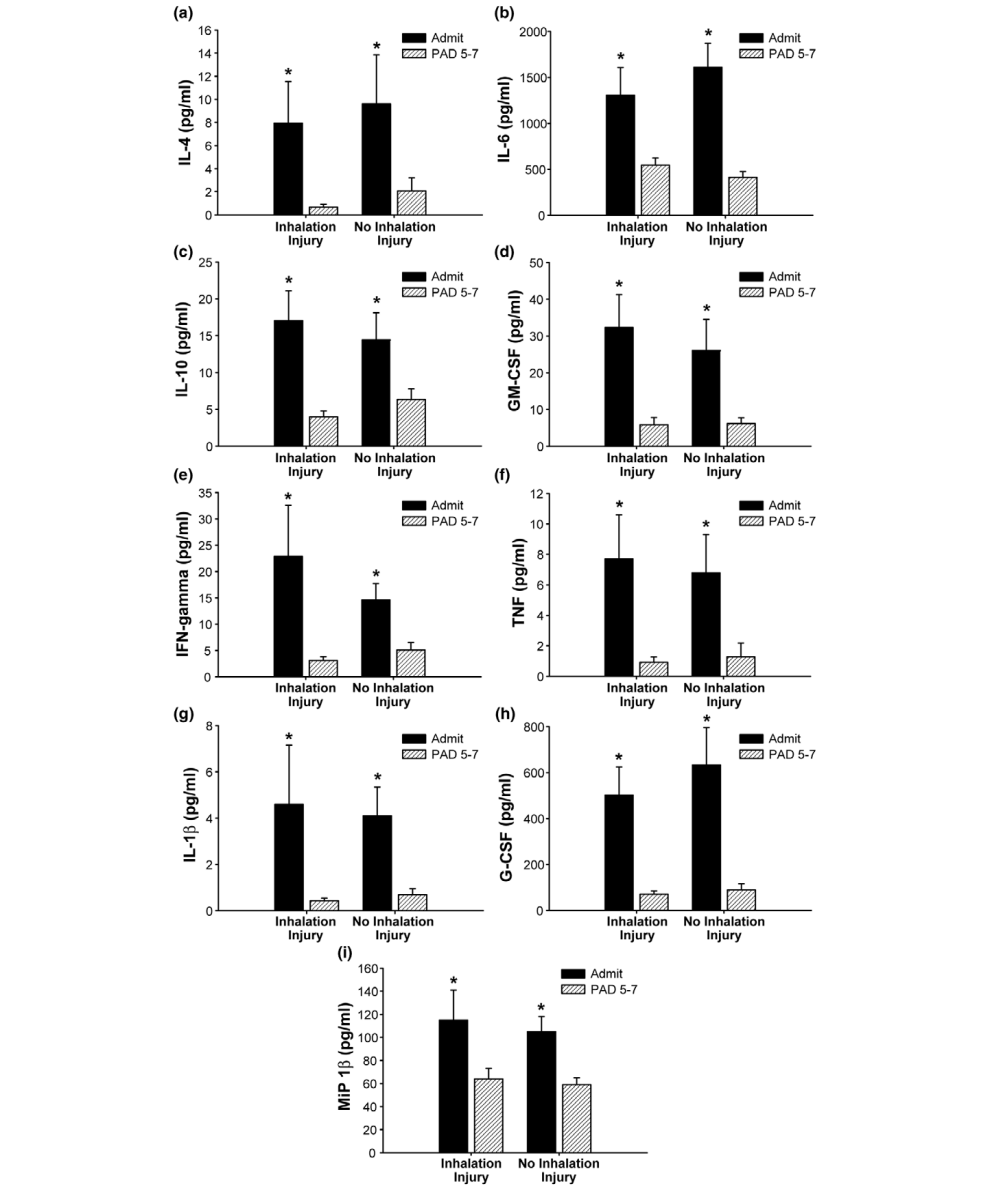
Cytokines presented demonstrated a significant decrease from admission to post-admission day (PAD) five to seven in the group with inhalation injury and the group with no inhalation injury. **(a) **Serum IL-4 (normal IL-4: 0 ± 0 pg/ml). **(b) **Serum IL-6 (normal IL-6: 8.7 ± 5 pg/ml). **(c) **Serum IL-10 (normal IL-10: 1.4 ± 0.3 pg/ml). **(d) **Serum GM-CSF (normal GM-CSF: 0 ± 0 pg/ml). **(e) **Serum IFN-γ (normal IFN-γ : 1.4 ± 0.5 pg/ml). **(f) **Serum TNF (normal TNF: 0.7 ± 0.007 pg/ml). **(g) **Serum IL-1β (normal IL-1β : 0.91 ± 0.007 pg/ml). **(h) **Serum G-CSF (normal G-CSF: 1.2 ± 1.2 pg/ml). **(i) **Serum MIP-1β (normal MIP-1β : 37 ± 9 pg/ml). *Significant difference between admit and PAD 5–7 (*p *< 0.05). Data are presented as mean ± standard error of the mean. GM-CSF, granulocyte-macrophage colony-stimulating factor; G-CSF, granulocyte colony-stimulating factor; IFN-γ, interferon-gamma; IL, interleukin; MIP-1β, macrophage inflammatory protein-1-beta; TNF, tumor necrosis factor.

**Figure 4:**
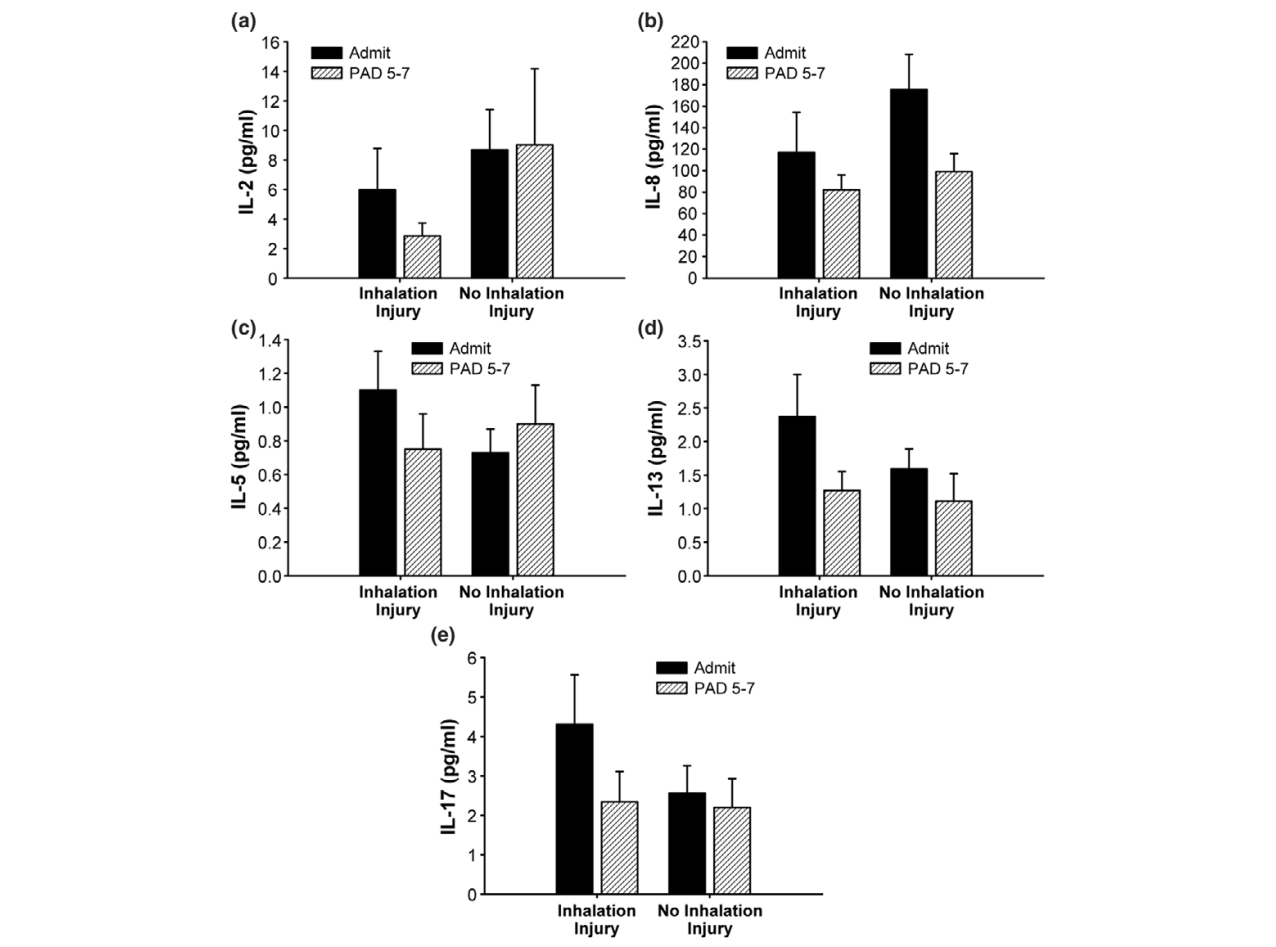
Cytokines presented were expressed at similar concentrations in regardless of inhalation injury status or time post burn. No significant differences or significant changes between admission and five to seven days after admission were found for **(a) **interleukin (IL)-2 (normal IL-2: 0 ± 0 pg/ml), **(b) **IL-8 (normal IL-8: 8 ± 5 pg/ml), **(c) **IL-5 (normal IL-5: 0.7 ± 0.14 pg/ml), **(d) **IL-13 (normal IL-13: 0.9 ± 0.2 pg/ml), and **(e) **IL-17 (normal IL-17: 0 ± 0 pg/ml). Data are presented as mean ± standard error of the mean. PAD, post-admission day.

**Figure 5:**
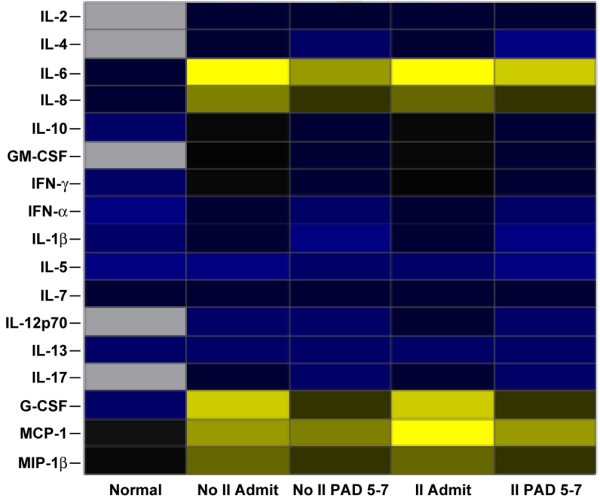
Heat map comparing serum cytokine protein expression profiles of non-burn (Normal), burn (No II Admit or No II PAD 5–7), and burn plus inhalation injury (II Admit or II PAD 5–7) groups. Values are log_10 _(average cytokine concentration, pg/ml) and blue indicates lower levels, yellow indicates highest levels, and black indicates levels in the middle. Gray squares indicate that no expression was detected. G-CSF, granulocyte colony-stimulating factor; GM-CSF, granulocyte-macrophage colony-stimulating factor; IFN, interferon; II Admit, inhalation injury group at admission; II PAD 5–7, inhalation injury group five to seven days after admission; IL, interleukin; MCP-1, monocyte chemoattractant protein-1; MIP-1β, macrophage inflammatory protein-1-beta; No II Admit, group with no inhalation injury, at admission; No II PAD 5–7, group with no inhalation injury, five to seven days after admission.

The concentrations of serum cytokines from 15 unburned, normal pediatric patients are included for comparison to the patients with inhalation injury and those without inhalation injury (Figures 2, 3, 4, 5). These data were previously published [10].

### Serum cytokines

Blood was collected at the time of admission and again five to seven days after admission. Blood was collected in serum-separator collection tubes. The tubes were centrifuged for 10 minutes at 1,320 rpm, and the serum was removed and stored at -70°C until assayed. Expression of 17 inflammatory mediators was measured using the Bio-Plex Human Cytokine 17-Plex panel with the Bio-Plex Suspension Array System (Bio-Rad Laboratories, Inc., Hercules, CA, USA). The following cytokines are detected simultaneously with this multi-plexed bead panel: IL-1β, IL-2, IL-4, IL-5, IL-6, IL-7, IL-8, IL-10, IL-12p70, IL-13, IL-17, granulocyte colony-stimulating factor (G-CSF), granulocyte-macrophage colony-stimulating factor (GM-CSF), interferon-gamma (IFN-γ), monocyte chemoattractant protein-1, macrophage inflammatory protein-1-beta (MIP-1β), and TNF. The assay was performed according to the manufacturer's instructions. Briefly, serum samples were thawed, centrifuged at 4,500 rpm for 3 minutes at 4°C, and incubated with microbeads labeled with antibodies specific to one of the aforementioned cytokines for 30 minutes. Following a wash step, the beads were incubated with the detection antibody cocktail, each bead specific to a single cytokine. After another wash step, the beads were incubated with streptavidin-phycoerythrin for 10 minutes and washed and then the concentrations of each cytokine were determined using the array reader (Figures 2, 3, 4).

### Ethics and statistics

The study was reviewed and approved by the Institutional Review Board of the University of Texas Medical Branch (Galveston, TX, USA). Prior to the study, each subject, parent, or child's legal guardian signed a written informed consent form. Two-tailed paired and unpaired Student *t *tests were used to compare differences in cytokine expressed. Data are expressed as percentages or mean ± standard error of the mean, where appropriate. Significance was accepted at a *p *value of less than 0.05.

## Results

Patient demographics are shown in Table 1. Children in the non-inhalation injury group showed similar age, gender, percentage of TBSA burned, percentage of third-degree burn, time from burn to admission, type of burn, and ethnicity compared to patients in the inhalation injury group. There was also no significant difference in length of hospital stay between the two groups (Table 1). We found that patients suffering from inhalation injury had a significantly higher mortality (43%; 13 of 30 patients) compared to burn patients without inhalation injury (12%; 5 of 42 patients) (*p *< 0.05).

**Table 1:** Demographics for patients with no inhalation injury and inhalation injury

	No inhalation injury	Inhalation injury
Number	42	30
Age in years	8 ± 1	9 ± 1
Female/male	12/30	13/17
Percentage of TBSA burned	60 ± 3	67 ± 4
Third-degree burn percentage	45 ± 3	56 ± 6
Time to admission in days	3 ± 0.3	2 ± 0.3
Length of hospital stay in days	35 ± 6	37 ± 4
Mortality, number (percentage)	5 (12%)	13 (43%)^a^

^a^Significant difference between control and inhalation injury groups (*p *< 0.05). TBSA, total body surface area.

All measured cytokine concentrations were significantly increased at the time of admission both in burned patients with inhalation injury and in those without inhalation injury compared to levels of non-burned, normal pediatric patients. By comparing patients suffering from a burn plus inhalation injury with patients suffering from a burn without inhalation injury, we found that expressions of only two cytokines were significantly different in the serum. Serum IL-7 was significantly higher in the non-inhalation injury group compared to the inhalation injury group (*p *< 0.05) (Figure 2a). Serum IL-12p70 was significantly lower in the non-inhalation injury group compared to patients with inhalation injury (*p *< 0.05) (Figure 2b). No other cytokines were significantly different between the two groups.

We found that many cytokines significantly decreased from admission to five to seven days after admission in both groups (Figure 3a–i). These cytokines include IL-4, IL-6, IL-10, GM-CSF, IFN-γ, TNF, IL-1β, G-CSF, and MIP-1β (*p *< 0.05). No significant differences between the groups or significant changes within the groups were found between admission and five to seven days after admission for serum IL-2, IL-8, IL-5, IL-13, and IL-17 (Figure 4). Cytokine profiles for the inhalation injured and non-inhalation injured patients were generated at the time of admission as well as five to seven days after admission and compared to profiles from normal patients (Figure 5). The heatmap shows that the cytokine response is not back to normal levels within this short post burn time period.

## Discussion

Of the injuries associated with burns, the single most important contributor to mortality is inhalation injury. Smoke inhalation-induced lung injury increases sloughing of ciliated epithelium, tracheal exudate production, and pulmonary capillary permeability [3]. Because lung permeability is increased soon after burn [4], resulting in accumulation of plasma in the lung, we hypothesized that an inhalation injury augments the inflammatory response and increases the systemic cytokine expression. However, we found that an inhalation injury does not augment the systemic inflammatory response but instead decreases IL-7 and increases IL-12p70 serum concentrations. The reason why these particular cytokines are modulated in response to inhalation injury is not known; however, we hypothesize that cytokines are locally consumed in the lung, thus lowering the overall systemic presence as measured in the serum. We further showed that important pro-inflammatory mediators known to be modulated in response to burn are not significantly different in patients with inhalation injury. We found that TNF, IL-6, and IL-8 all major inflammatory mediators were not significantly different. Similar results were found by Hales and colleagues [20] in an animal model, in which the authors showed that TNF does not change with inhalation injury. We have confirmed that TNF is not a major mediator after inhalation injury.

Another animal study investigated the effect of inhalation injury on alveolar macrophages [21]. The authors found that smoke-exposed macrophages and inhalation of smoke suppressed both alveolar macrophage adherence to plastic and phagocytosis of opsonized bacteria. Basal superoxide production was elevated whereas basal secretion of TNF was suppressed. The authors concluded that the early responses of alveolar macrophages to smoke inhalation lung injury consist of a functional downregulation of phagocytosis.

In the present study of 72 patients, we found two discrete cytokines that were significantly different in patients suffering from inhalation injury versus patients with no inhalation injury, IL-7 and IL-12p70. IL-7 was significantly higher in the patients without inhalation injury. IL-7 is critical for regulating lymphoid homeostasis and has been shown to be critical for the differentiation of most T cells. Studies in IL-7 knockout mice showed that IL-7 has anti-apoptotic effects on T cells via Bcl-2 expression, indicating that IL-7 plays an important role in T-cell regulation and homeostasis [22]. IL-12p70 was significantly increased in patients with inhalation injury. IL-12p70 has IL-7-like effects on T-cell function and maturation but its role is not as clearly understood [23]. In a recent study, it was shown that mouse splenic CD8α^+ ^and CDα^-^CD4^- ^dendritic cells (DCs) had the ability to produce either IL-12p70 or IL-10 depending on the nature of the pathogen encountered. In contrast, CD4^+ ^DCs seem incapable of producing IL-12p70 [24]. The exact role of IL-7 and IL-12p70 in the pathophysiologic cascade following burn with smoke inhalation needs to be determined.

We hypothesized that inhalation injury increases mortality by increasing the systemic inflammatory response soon after burn. The systemic inflammatory response after a severe burn injury leads to hypermetabolism and to protein degradation. Consequently, the structure and function of essential organs such as the muscle, skin, heart, immune system, and liver are compromised, contributing to multi-organ failure and mortality [15,18]. It has been suggested that uncontrolled release of pro-inflammatory mediators triggers and enhances protein wasting and organ dysfunction [25,26]. Organ function breakdown can then lead to increased incidence of infection and sepsis, ultimately leading to multi-organ failure and death. Our results indicate that inhalation injury does not cause an augmented inflammatory response. Thus, the mechanism by which inhalation injury causes an increase in mortality is not the systemic inflammatory response soon after burn. Based on these results, we will determine the hypermetabolism and energy expenditure in burned children with and without inhalation injury in order to find the cause for the increased mortality in burned children with inhalation injury.

A very important aspect that was outside the scope of this study is the determination of cytokine levels in the lung. Because we hypothesize that cytokines are locally increased and that this causes the systemic cytokines to be decreased, we have to perform a follow-up study in which we correlate local (lung) cytokine concentrations in the bronchoalveolar lavage fluid to the levels of cytokines in the blood.

## Conclusion

Inhalation injury is a major contributor to mortality in severely burned patients. The molecular and cellular mechanisms by which smoke inhalation causes this increase are not known. We hypothesized that inhalation injury augments the inflammatory response and subsequently increases the hypermetabolism and catabolism leading to multi-organ failure and death. This was not the case, and our hypothesis was disproven. In the present study, we showed that a severe burn with an inhalation injury causes decreased IL-7 and increased IL-12p70 cytokine levels at the time of admission to the hospital, but these differences disappear following five to seven days after admission. Because the altered cytokines play an important role in the immune system and host defense, we suggest that instead of an augmented systemic inflammatory response soon after burn, immunocompromise and immunodysfunction may be involved in the increased mortality in patients suffering from burns plus inhalation injury.

## Key messages

• Inhalation injury does not cause an augmented inflammatory response.

• Inhalation injury interferes with important T-cell mediators.

## Abbreviations

DC = dendritic cell; GM-CSF = granulocyte-macrophage colony-stimulating factor; IFN-γ = interferon-gamma; IL = interleukin; MIP-1β = macrophage inflammatory protein-1-beta; TBSA = total body surface area; TNF = tumor necrosis factor.

## Competing interests

The authors declare that they have no competing interests.

## Authors' contributions

CCF was responsible for planning the study, collecting data, running analysis, and writing the manuscript. DNH was responsible for patient care and collecting specimens, collecting data, analysis, and manuscript preparation. MGJ was responsible for planning the study, patient care and collecting specimens, data analysis and statistics, and writing the manuscript. All authors read and approved the final manuscript.
